# Organellar Genome Analysis of the Red Alga *Rhodymenia intricata* (Rhodophyta, Florideophyceae) and Its Phylogenetic Analysis

**DOI:** 10.3390/life16091391

**Published:** 2026-08-24

**Authors:** Maheshkumar Prakash Patil, Yong Jun Park, Jeong Woo Cho, Kwangsup Lee, Shin-Ichi Kitamura, Ganesh Bansi Patil, Rakesh Eshwarlal Mutha, Jong-Oh Kim, Kyunghoi Kim

**Affiliations:** 1 Department of Life Science, Parul Institute of Applied Sciences, Parul University, Vadodara 391760, Gujrat, India; maheshkumar.patil46404@paruluniversity.ac.in; 2 School of Marine and Fisheries Life Science, Pukyong National University, 45 Yongso-ro, Nam-Gu, Busan 48513, Republic of Korea; 3 Marine Eco-Technology Institute, Busan 48520, Republic of Korea; kslee@marine-eco.co.kr; 4 Graduate School, Faculty of Bioresources, Mie University, 1577 Kurimachiya-cho, Tsu City 514-8507, Mie Prefecture, Japan; kitamura@bio.mie-u.ac.jp; 5 Department of Quality Assurance, H. R. Patel Institute of Pharmaceutical Education and Research, Shirpur 425405, Maharashtra, India; ganesh.patil@hrpatelpharmacy.co.in; 6 Department of Pharmacognosy, H. R. Patel Institute of Pharmaceutical Education and Research, Shirpur 425405, Maharashtra, India; rakesh.mutha@hrpatelpharmacy.co.in; 7 Department of Microbiology, Pukyong National University, 45 Yongso-ro, Nam-Gu, Busan 48513, Republic of Korea; 8 Department of Ocean Engineering, Pukyong National University, 45 Yongso-ro, Nam-Gu, Busan 48513, Republic of Korea

**Keywords:** *Rhodymenia intricata*, mitochondrial genome, phylogenetic analysis, Rhodymeniales, codon usage bias, red algae

## Abstract

Red algae (Rhodophyta) are an ancient lineage of photosynthetic eukaryotes that play important roles in marine ecosystems. However, genomic information for many species within the order Rhodymeniales remains limited, particularly for mitochondrial genomes. In this study, we sequenced, assembled, and analyzed the complete mitochondrial genome (mitogenome) of *Rhodymenia intricata* to investigate its genome organization, gene content, and phylogenetic position within Rhodymeniales. The mitogenome of *R. intricata* is a circular DNA molecule of 26,213 bp containing 49 genes, including 25 protein-coding genes (PCGs), 21 tRNA genes, and 3 rRNA genes. The genome shows a strong A + T bias (70.9%) and positive AT and GC skews, typical of red algal mitogenomes. Comparative analysis with other Rhodymeniales mitogenomes revealed generally conserved gene content and organization, with several lineage-specific features such as the presence of the *rpl20* gene, an additional open reading frame (*orf148*), and three rRNA genes (*rnl*, *rns*, and *rns5*). Codon usage analysis indicated a preference for leucine and isoleucine codons and dominant start and stop codons (ATG and TAA). Phylogenetic analysis based on a concatenated dataset of 23 mitochondrial PCGs strongly supported the monophyly of Rhodymeniales and confirmed the close relationship between *R. intricata* and *R. pseudopalmata*. Overall, this study presents the first complete mitogenome of *R. intricata* and expands mitogenomic resources for Rhodymeniales, providing new insights into mitogenome evolution and phylogenetic relationships in red algae.

## 1. Introduction

Red algae (phylum Rhodophyta) are among the most ancient photosynthetic eukaryotes and play key roles in marine ecosystems by driving primary production, forming benthic habitats, and supporting ecological diversity [1,2]. The class Florideophyceae contains most extant red algal diversity, with morphologically complex taxa across temperate and tropical seas [3]. Within this class, the order Rhodymeniales is ecologically important, exhibiting diverse reproductive traits and thalli ranging from fleshy to flattened or hollow [4]. Despite their prominence, genomic data, particularly organellar genomes, remain limited. The genus *Rhodymenia* (Rhodymeniaceae) includes widely distributed species inhabiting rocky intertidal and subtidal zones. *Rhodymenia intricata* (Okamura) Okamura 1930, found mainly in the Northwest Pacific [5], is well-documented morphologically but lacks comprehensive genomic studies. Ecologically, *R. intricata* contributes to habitat complexity and nutrient cycling in coastal environments. Additionally, species of *Rhodymenia* are of applied interest due to their potential as sources of bioactive compounds and as food in some regions. In particular, the absence of complete mitochondrial genome (mitogenome) data has restricted phylogenetic analyses within Rhodymeniales.

Mitogenomes are valuable for phylogenetics due to their conserved gene content and compact structure. In red algae, they typically encode oxidative phosphorylation genes (*nad*, *cox*, *atp*, *sdh*), ribosomal proteins, rRNAs, and tRNAs. While gene content is broadly conserved, variations in genome size, nucleotide composition, introns, and gene order occur across lineages [6,7,8], offering insights into genome evolution beyond morphology. Next-Generation Sequencing has enabled rapid assembly of organellar genomes in red algae [9]. Yet, mitogenomes for Rhodymeniales remain scarce, limiting comparative studies. Expanding these resources can reveal structural conservation, codon usage, and nucleotide composition patterns, enhancing understanding of mitochondrial evolution [8,10]. Mitochondrial genomes often provide higher resolution for phylogenetic analyses in red algae due to their conserved gene content, uniparental inheritance, and reduced recombination compared to nuclear markers, making them especially valuable for resolving evolutionary relationships where nuclear data may be ambiguous.

Phylogenetic analyses based on concatenated mitochondrial protein-coding genes (PCGs) have proven effective for resolving evolutionary relationships among red algae at both intergeneric and interspecific levels [11,12,13]. Model-based approaches, such as maximum likelihood methods, enable robust reconstruction of phylogenetic relationships and assessment of statistical support for inferred clades. For Rhodymeniales, mitogenome-based phylogenies can clarify interspecific relationships and refine the systematic framework of the order, addressing challenges posed by morphological plasticity and overlapping diagnostic traits.

This study reports the whole mitogenome sequence of *Rhodymenia intricata* and presents a comprehensive analysis of its genomic architecture. The mitogenome was assembled and annotated to identify PCGs, tRNAs, rRNAs, and structural features. We further conducted comparative analyses with other available mitogenomes within Rhodymeniales to evaluate genome structure, nucleotide composition, and gene content conservation. In addition, phylogenetic reconstruction based on concatenated mitochondrial PCGs was performed to determine the evolutionary position of *R. intricata* within the order. This study expands mitogenomic resources for Rhodymeniales and provides new insights into mitogenome evolution and phylogenetic relationships within this ecologically important group.

## 2. Materials and Methods

### 2.1. Sample Collection and DNA Extraction

**Figure 1:**
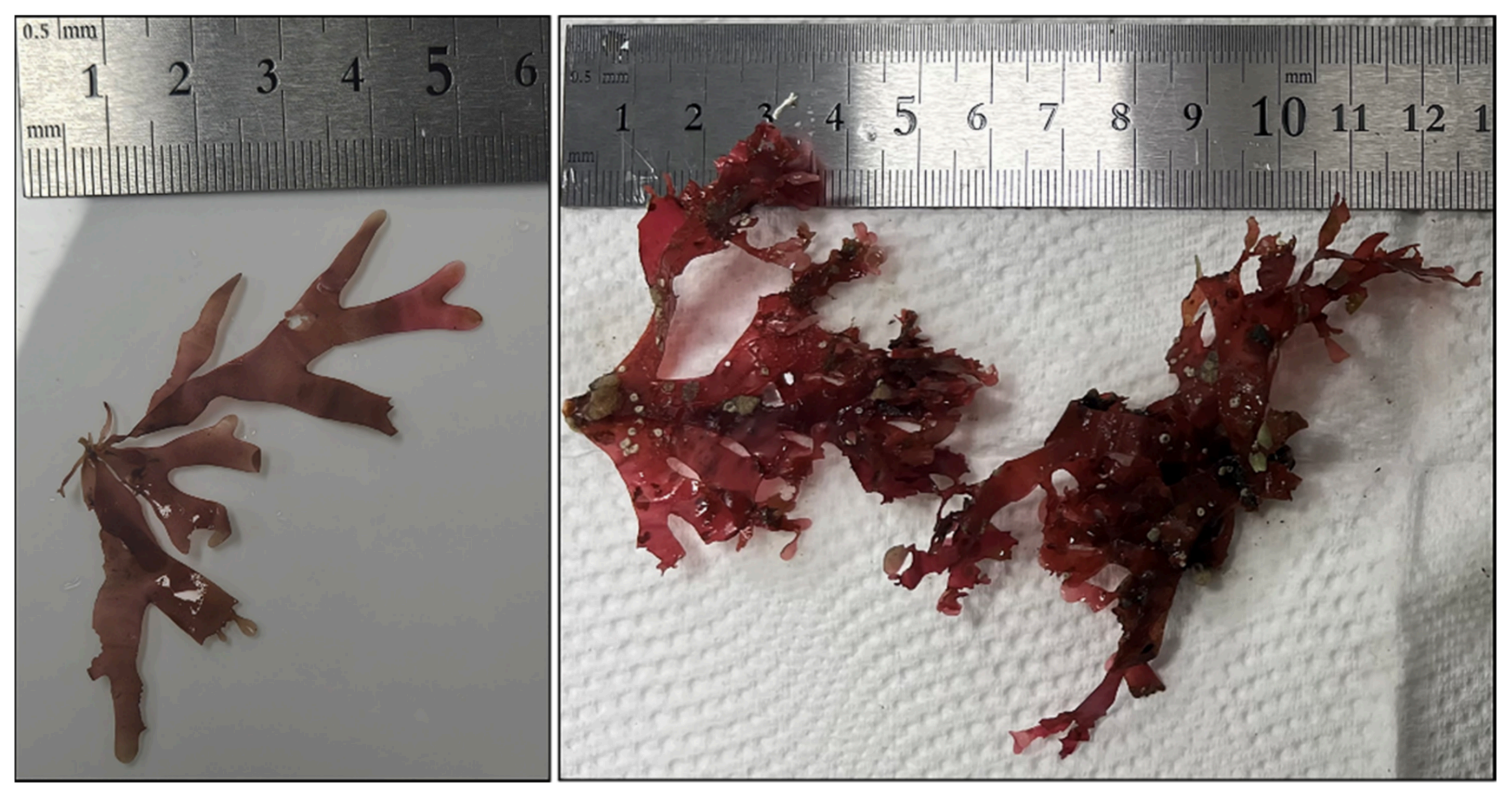
A specimen of *Rhodymenia intricata*, a macroalga collected from the East Sea near Busan, South Korea. The thallus measures 2–6 cm in length, is bright red in color, and leaves are flat and fan-shaped. Branching is irregular, and the branch tips are rounded.

A specimen of the red macroalga *Rhodymenia intricata* (Figure 1) was collected in the coastal waters of Busan, South Korea (35°16′ N, 129°15′ E) in August 2024. This sample is currently archived at the Ecological Restoration Group of the Marine Eco-Technology Institute (Busan) with ID PU-T01-S-MA-11. Total genomic DNA was extracted with the DNeasy Plant Kit (Qiagen, Hilden, Germany) according to the manufacturer’s instructions. The DNA concentration was quantified using a NanoDrop spectrophotometer (Thermo Fisher Scientific, D1000, Waltham, MA, USA) to ensure quality for downstream applications. Purified DNA extracts are kept at −20 °C.

### 2.2. Library Preparation, Sequencing, and Mitogenome Assembly

Genomic DNA was prepared using the TruSeq Nano DNA Library Preparation Kit (Illumina, San Diego, CA, USA) and sequenced on the Illumina HiSeq 2500 platform by Macrogen Inc. (Daejeon, South Korea; https://www.macrogen.com/ko/, accessed on 20 August 2026). Paired-end sequencing (2 × 150 bp) was performed, targeting an average insert size of 350 bp. To improve data quality, low-quality reads (Phred score < 20) and adapter sequences were removed using Trimmomatic v0.38 [14]. Read quality was evaluated with FastQC v0.11.7 [15]. A subset of high-quality reads was randomly selected for de novo mitogenome assembly. The mitogenome of *R. intricata* was assembled using SPAdes v3.15.0 [16] based on Illumina short-read data. The mitogenome of *Rhodymenia pseudopalmata* (GenBank accession: NC_023252.1), a representative species of the Rhodymeniaceae, was used as the seed and reference sequence.

### 2.3. Mitogenome Annotation and Analysis

The mitogenome (assembled) was compared with previously published mitogenome sequences in the NCBI database using BLAST (version 2.17.0). Genome annotation was conducted using MFannot [17] with Translation Table 4. PCGs were predicted using ORF Finder and subsequently validated through BLAST searches against the NCBI protein database [18]. RNA genes were identified using RNAmmer [19] and tRNAscan-SE 2.0 [20], while RNAweasel was employed to confirm RNA gene boundaries and detect potential intronic regions [21]. Tandem repeats were detected using Tandem Repeats Finder v4.09 [22]. A circular mitogenome map was generated using OGDraw v1.3.1 [23]. MEGA11 (v11.2.8) was used to determine the nucleotide composition and relative synonymous codon usage (RSCU) [24]. Strand asymmetry was assessed according to the formulas GC-skew = (G − C)/(G + C) and AT-skew = (A − T)/(A + T) [25].

### 2.4. Phylogenetic Analysis

To determine the phylogenetic position of *R. intricata* within the order Rhodymeniales, a phylogenetic analysis was conducted based on mitochondrial PCG sequences. A total of five complete mitogenomes, including that of *R. intricata*, were retrieved from the GenBank database according to the updated phylogenetic framework of the order Rhodymeniales [12]. Four species were designated as the ingroup, whereas *A. flabellatum* was selected as the outgroup (Table 1). The analysis included 23 conserved mitochondrial PCGs: seven NADH dehydrogenase subunits (*nad1-6* and *nad4 L*), four ATP synthase subunits (*atp4*, *atp6*, *atp8*, and *atp9*), four ribosomal proteins (*rps3*, *rps11*, *rps12*, and *rpl16*), three cytochrome c oxidase subunits (*cox1-3*), three succinate dehydrogenase subunits (*sdh2-4*), apocytochrome b (*cob*), and the secY-independent protein translocase (*tatC*). Nucleotide sequences of these genes were concatenated and aligned using MAFFT v7.0 [26]. The best-fitting nucleotide substitution model was selected using ModelFinder implemented in IQ-TREE, based on the Bayesian Information Criterion (BIC), which identified GTR + F + I + G4 as the optimal model [27]. A Maximum Likelihood (ML) phylogenetic tree was constructed in IQ-TREE, and node reliability was assessed with 1000 ultrafast bootstrap replicates [28]. Branch support was evaluated with 1000 ultrafast bootstrap replicates to assess node reliability. The resulting ML tree was visualized using the Interactive Tree of Life (iTOL) v7 web interface [29].

## 3. Results

### 3.1. Genome Structure, Organization and Composition

**Figure 2:**
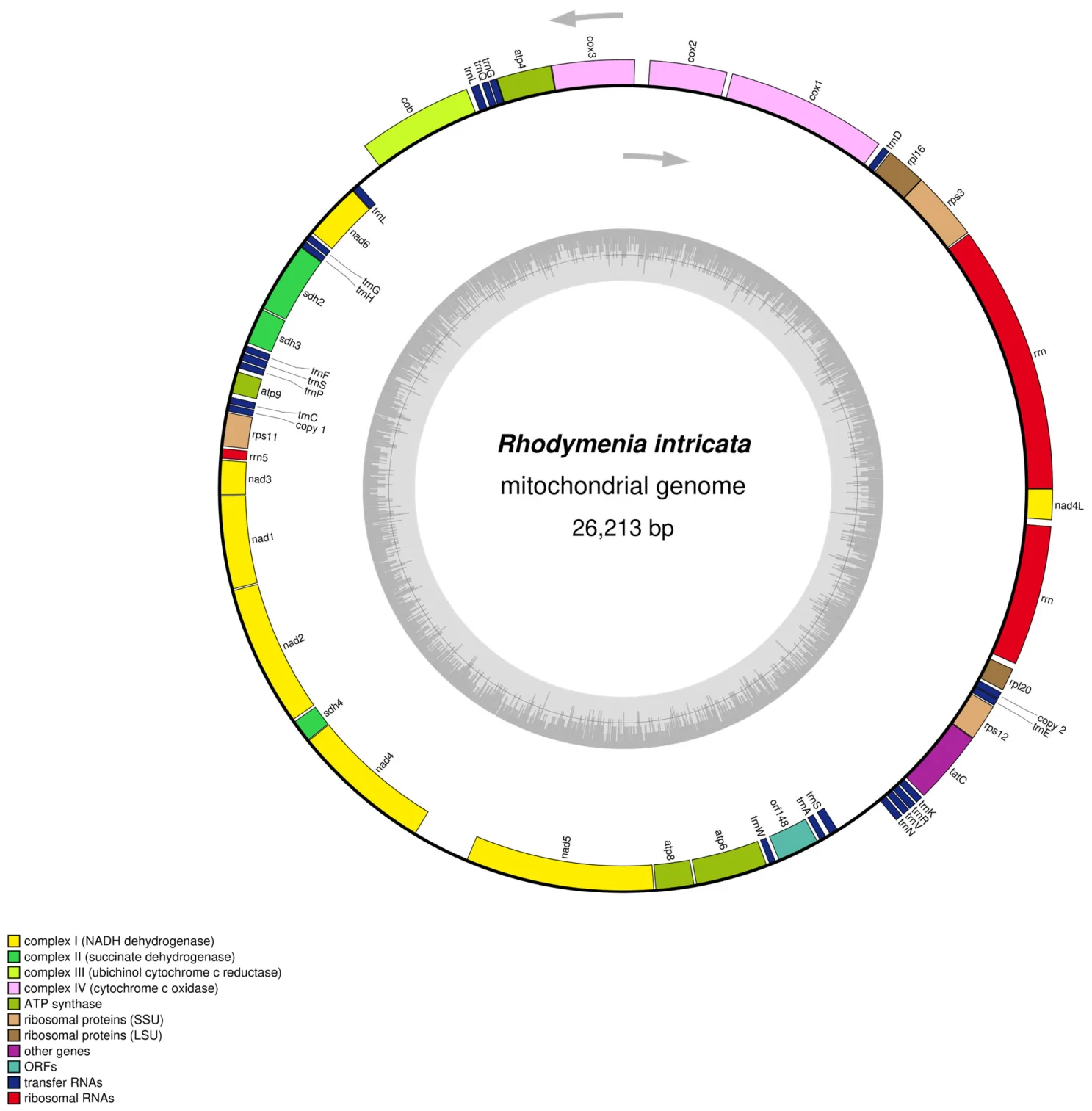
Circular mitochondrial genome of *Rhodymenia intricata* (GenBank accession number PX733923). Arrows indicate gene orientation, and colors represent functional gene groups with their corresponding acronyms.

Sequencing of the *R. intricata* genomic library using the Illumina platform generated 16,023,654 raw reads with a GC content of 43.7%. During preprocessing, duplicate reads and potential contaminants were also removed to ensure high-quality input for assembly. The data showed high quality, with Q20 and Q30 scores of 98.3% and 94.1%, respectively. After filtering low-quality reads, 14,910,404 clean reads were retained, with improved quality scores (Q20: 99.6%; Q30: 96.7%) and a GC content of 43.2%. De novo assembly of the filtered reads achieved complete mitogenome coverage with an average depth of 43.65 X. The average insert size was 375.65 bp (±135.57 bp). Sequencing depth was assessed across the mitogenome and found to be evenly distributed, supporting the reliability of the assembly. Circularization of the contig was validated by confirming overlapping terminal sequences and consistent read mapping across the junction. The final assembly produced a single circular contig of 26,213 bp, representing the complete mitogenome of *R. intricata* (Figure 2), deposited in GenBank under accession number PX733923. The mitogenome GC content was 29.1%, markedly lower than that of the raw reads. Annotation identified 49 genes, including 25 PCGs, 21 tRNAs, and 3 rRNAs (Table 2). One open reading frame (ORF), *orf148*, was also detected. The heavy (H) strand encoded 11 PCGs, 10 tRNAs, and 2 rRNAs, whereas the light (L) strand encoded 14 PCGs (including *orf148*), 11 tRNAs, and 1 rRNA. The mitogenome showed a strong A + T bias (70.9%), with nucleotide proportions of 37.4% A, 33.6% T, 14.7% G, and 14.4% C (Table 3). Positive AT skew (0.054) and GC skew (0.009) values indicate a slight preference for A over T and G over C.

### 3.2. Intergenic Regions and Codon Usage

**Figure 3:**
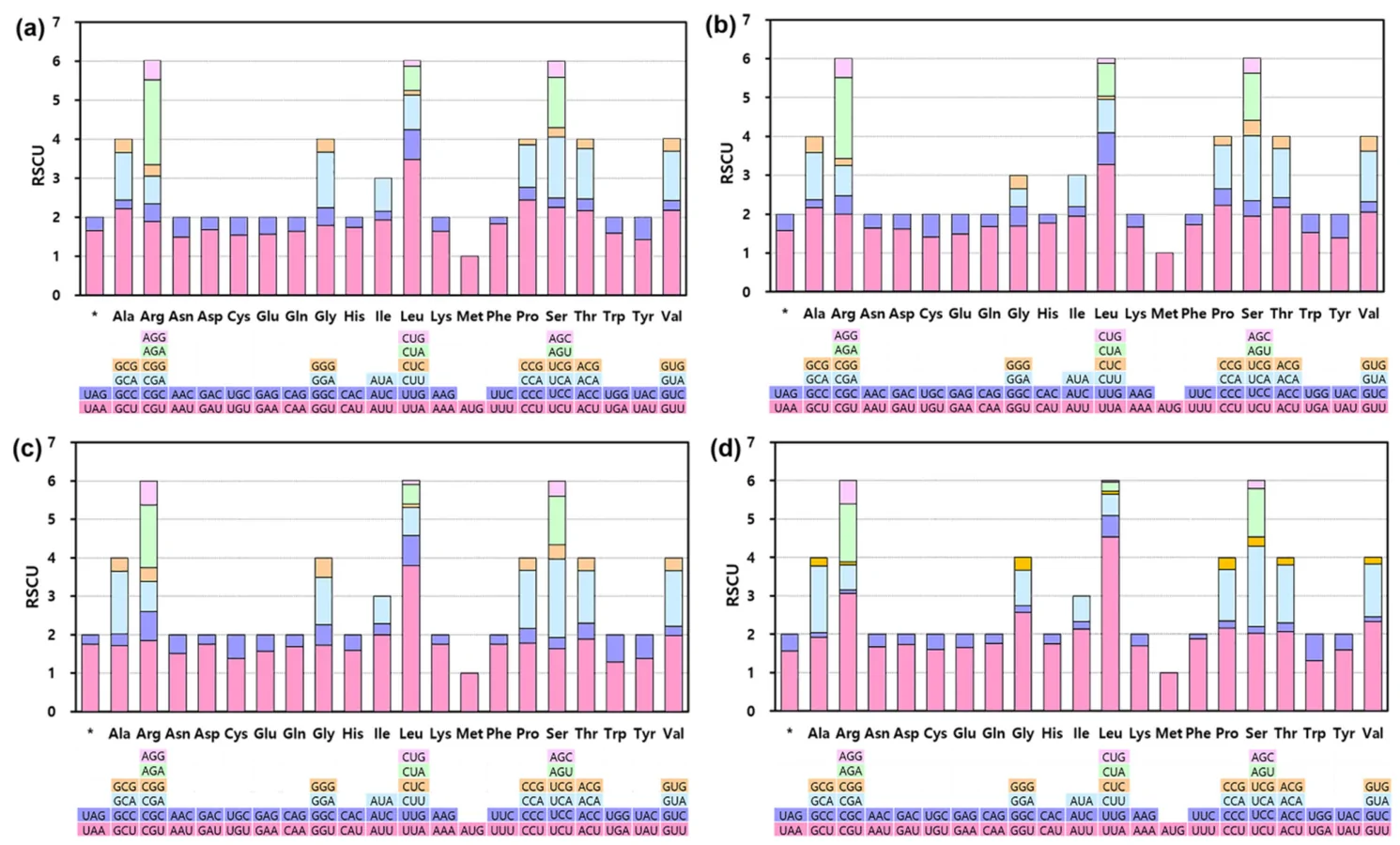
Relative synonymous codon usage (RSCU) patterns of mitochondrial PCGs across four Rhodymeniales species: (**a**) *Rhodymenia intricata*, (**b**) *Rhodymenia pseudopalmata*, (**c**) *Fushitsunagia catenata*, and (**d**) *Coeloseira compressa*. Note: * indicate stop codon.

Analysis of the mitogenome of *R. intricata* revealed only one gene overlap, consisting of a single base pair between the *rps3* and *rpl16* genes. All other genes were separated by intergenic spacer regions ranging from 1 to 597 bp. In total, these spacers covered 2395 bp, accounting for 9.1% of the mitogenome. The longest spacer (597 bp) was located between *nad4* and *nad5* (Table 2). RSCU analysis of the mitochondrial PCGs was performed and compared with three other Rhodymeniales species (Figure 3). The PCGs encoded 5798 amino acids. Leucine was the most abundant (853 residues; 14.7%), followed by isoleucine (10.9%), phenylalanine (9.7%), serine (8.9%), and valine (5.9%). A clear codon usage bias was observed. The codon UUA (leucine) was the most frequently used (493 occurrences) and had the highest RSCU value (3.47), indicating strong preference. AUG (methionine) showed an RSCU value of 1, suggesting no bias. Overall, 29 codons had RSCU values above one (preferred), while 34 had values below one, indicating lower usage.

### 3.3. Protein Coding Genes

The mitogenome of *R. intricata* contains 25 PCGs covering 18,069 bp, which represents 68.9% of the total genome. The overall AT content of these genes is 71.3% (Table 3). Table 4 summarizes the base skew values and nucleotide composition for all genes. Among the PCGs, *nad5* is the longest gene (1986 bp), and *rpl20* is the shortest (228 bp). All genes show a strong A + T bias, with AT content ranging from 64.5% (*atp9*) to 77.3% (*atp4*). Strand asymmetry analysis showed that most genes have negative AT-skew values, indicating more thymine than adenine. However, four genes (*rpl16*, *rps11*, *rps12*, and *rpl20*) showed positive AT-skew. For GC-skew, most genes were positive, while eleven genes (*rps3*, *rpl16*, *atp4*, *sdh2*, *sdh3*, *rps11*, *atp8*, *atp6*, *orf148*, *tatC*, and *rps12*) had negative values (Table 4). Comparison with other Rhodymeniales species shows that most possess 24–26 PCGs, indicating strong conservation of mitochondrial gene content (Table 5). ATG was the most common start codon and TAA the most frequent stop codon. Exceptions included *cox2*, *rps11*, and *tatC* (TTG start codon) and *cox2*, *sdh2*, *nad4*, and *nad5* (TAG stop codon). The mitogenome also contains one ORF gene (*orf148*).

### 3.4. Transfer and Ribosomal RNA Genes

Most mitogenomes of Rhodymeniales contain two rRNA genes forming the typical rRNA operon (Table 1). In contrast, the mitogenome of *R. intricata* contains three rRNA genes (Table 2; Figure 2). Two genes, *rnl* (2654 bp) and *rns* (1388 bp), are located on the H-strand, while *rns5* (107 bp) is found on the L-strand. Together, these rRNA genes span 4149 bp, accounting for 15.8% of the mitogenome. The *rnl* and *rns* genes are separated by *nad4 L*, and *rns5* is positioned between *rps11* and *nad3*. A total of 21 tRNA (Table 2) genes were identified, covering 1600 bp (6.1% of the genome). Their lengths range from 72 bp (*trnD*, *trnG*, *trnW*) to 94 bp (*trnS*). No introns were detected, and *trnI* was not found in the mitogenome. Anticodon analysis showed standard codon–anticodon pairing patterns, similar to other Rhodymeniales species. Three tRNA genes (*trnG*, *trnL*, and *trnS*) each had two different anticodons, whereas methionine had only one. This pattern suggests some redundancy and flexibility in mitochondrial codon recognition in *R. intricata*.

### 3.5. Phylogeny

**Figure 4:**
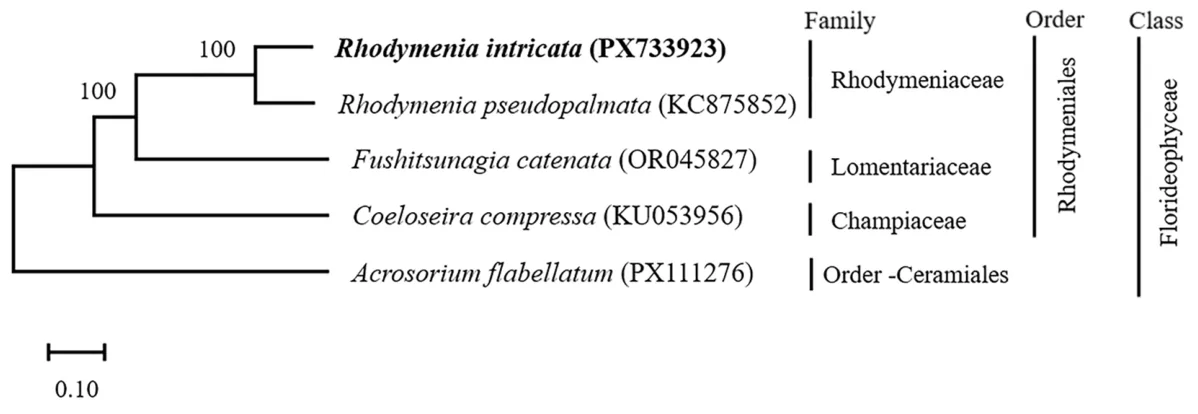
ML phylogenetic tree of the order Rhodymeniales constructed from a concatenated alignment of 23 conserved PCGs. The tree was rooted with *Acrosorium flabellatum* (PX111276) as the outgroup. Bootstrap support values (%) are shown at each node. Tree visualized using iTOL. Newly generated sequence from this study is indicated in bold. GenBank accession numbers are provided in parentheses following the species names.

An ML phylogenetic tree was constructed using a concatenated dataset of 23 conserved PCGs. The phylogenetic tree was rooted using *A. flabellatum* as the outgroup (Figure 4). The resulting topology clearly resolved the evolutionary relationships among the sampled Rhodymeniales taxa and showed strong statistical support at most nodes. The two species, *R. intricata* and *R. pseudopalmata,* clustered together with high bootstrap support, confirming their close relationship within the family Rhodymeniaceae. In contrast, *F. catenata* and *C. compressa* formed separate and well-defined branches, consistent with their classification in different families. The key nodes showed bootstrap values of 100%, indicating very strong confidence in the inferred relationships. The newly generated mitogenome sequence in this study was clearly positioned within the Rhodymeniales clade, supporting its taxonomic placement. Overall, the phylogenetic tree agrees with current family-level classifications and demonstrates that concatenated mitochondrial PCGs provide reliable markers for resolving evolutionary relationships within Rhodymeniales.

## 4. Discussion

The mitogenome of *R. intricata* shows many features typical of Rhodymeniales. It has a circular structure, biased AT composition, positive AT and GC skews, and a compact organization. We did not compare total gene counts with *C. compressa* (KU053956) because only a partial mitogenome sequence is available for this species [31]. The genome size of *R. intricata* (26,213 bp) is slightly larger than most other Rhodymeniales mitogenomes (Table 1). The observed increase is primarily due to the presence of the *rpl20* gene, an additional *orf148* gene (Table 2), and the overall greater cumulative length of PCGs (Table 3).

The positive AT and GC skew values indicate strand-specific nucleotide preferences, a pattern also reported in other Rhodymeniales species [12,30,31]. Such compositional asymmetry reflects lineage-specific evolutionary pressures acting on mitogenomes. Intergenic regions in *R. intricata* occupy only a small portion of the genome and include a single gene overlap and short intergenic spacers (Table 2). The complete absence of introns in the *R. intricata* mitogenome reflects mitochondrial genome streamlining, a characteristic feature of many Florideophyceae that likely results from ancestral intron loss and/or reduced intron invasion. This compact gene arrangement aligns with patterns observed in other red algal mitogenomes, where genome expansion typically arises through gene duplication or intron insertion rather than the accumulation of large non-coding regions [8,9,32]. Codon usage analysis revealed a bias toward specific amino acids, particularly leucine and isoleucine, and a clear preference for ATG as the start codon and TAA as the stop codon (Figure 3; Table 5). Similar codon usage patterns have been reported in other Rhodymeniales mitogenomes, suggesting that translational selection plays an important role in shaping codon composition in this group [12,30]. Overall, the PCG content of *R. intricata* is largely conserved within Rhodymeniales, but some lineage-specific differences are evident.

One notable feature is the presence of the *rpl20* gene and an additional ORF (*orf148*), which are absent in *R. pseudopalmata* and *F. catenata* (Table 5) [12,30]. Gain and loss of genes such as *rpl20*, *tatC*, and *sdh* have been documented in other red algal lineages, reflecting the dynamic nature of mitogenome evolution [9,33]. These lineage-specific changes may result from functional shifts, gene transfer to the nucleus, or mitochondrial–nuclear coevolution [9,34]. Despite these differences, the overall PCG composition and nucleotide bias patterns remain highly conserved across Rhodymeniales (Table 3), highlighting strong evolutionary constraints on mitochondrial function.

The origin of *orf148* may represent a relatively recent genomic finding. Novel mitochondrial ORFs can arise through several mechanisms, including local sequence rearrangements, duplication followed by sequence divergence, or accumulation of mutations within previously non-coding regions that create a new uninterrupted open reading frame [35,36]. In compact mitochondrial genomes, where intergenic regions are often limited, the emergence of a novel ORF may result from the modification or repurposing of pre-existing genomic sequences [37]. At present, however, there is insufficient evidence to determine whether orf148 represents a functional protein-coding gene or a non-functional ORF. In particular, the absence of experimental evidence for transcription, translation, or a conserved protein domain prevents assignment of a specific biological function [38]. Comparative analysis of related red algal mitochondrial genomes may therefore be useful for determining whether *orf148* is lineage-specific or represents a more widely conserved feature [39].

The identification of TTG as the predicted start codon for *cox2*, *rps11*, and *tatC* in *R. intricata* provides an interesting feature of its compact mitogenome. Although AUG is the canonical translation-initiation codon, alternative initiation codons such as UUG (TTG in the DNA sequence) can be recognized by translation systems of bacterial and organellar origin [40,41,42]. Thus, these TTG sites may represent functional alternative initiation codons rather than annotation errors [42]. Their occurrence in three mitochondrial genes suggests that the mitochondrial translation machinery of *R. intricata* may tolerate some flexibility in start-codon recognition. Such flexibility could reflect the evolutionary history of mitochondria and the retention of bacterial-type translational features despite extensive genome reduction [43].

The rRNA and tRNA gene complement of *R. intricata* is also generally conserved. Its mitogenome contains three rRNA genes (*rns*, *rnl*, and *rns5*), whereas *R. pseudopalmata, F. catenata* and *C. compressa* lack the *rns5* gene [12,30,31]. The presence of 21 tRNA genes in *R. intricata* is similar to *R. pseudopalmata*, but slightly fewer than the 19 tRNAs reported for *C. compressa*, and slightly lower than the 23 tRNAs reported for *F. catenata* (Table 1). In Rhodymeniales, the number of tRNAs typically ranges from 18 to 23 [9,12,44]. Interestingly, the *trnI* gene is absent in *R. intricata*, *F. catenata*, and *C. compressa*, but present in *R. pseudopalmata* [12,30,31]. Previous studies have shown that reanalysis of red algal mitogenomes with updated sequencing and annotation tools sometimes reveals genes previously considered lost [9], suggesting that careful reannotation remains important.

The presence of the *rns5* gene is another notable feature of the *R. intricata* mitochondrial genome. The *rns5* gene encodes a component of the small subunit of the mitochondrial ribosome and is therefore associated with the machinery required for mitochondrial protein synthesis [45,46]. The presence of *rns5* in *R. intricata* appears to represent a lineage-specific feature rather than a universally conserved characteristic across Rhodophyta [6,8].

The absence of the mitochondrial *trnI* gene in *R. intricata* is notable because isoleucine represents approximately 10.9% of the amino acid composition of its mitochondrial PCGs. The relatively high requirement for isoleucine suggests that its incorporation during mitochondrial protein synthesis must nevertheless be efficiently maintained. One possible mechanism is the import of nuclear-encoded tRNAs into mitochondria [47,48]. In several eukaryotic organisms, loss of mitochondrial tRNA genes has been compensated by the import of corresponding tRNAs synthesized from nuclear genes [42,49]. Thus, nuclear-encoded isoleucine tRNA import may potentially compensate for the loss of mitochondrial *trnI* in *R. intricata* [50]. However, this hypothesis cannot be confirmed from the mitogenome sequence alone. The possibility of an unrecognized divergent mitochondrial tRNA or other lineage-specific mechanisms of tRNA recognition also cannot be excluded. Comparative nuclear genomic and transcriptomic analyses, together with direct characterization of mitochondrial tRNAs, will be required to determine how isoleucine decoding is maintained in *R. intricata* [49].

Although anticodon usage is generally conserved, the presence of multiple anticodon variants for certain tRNAs (*trnG*, *trnL*, and *trnS*) indicates functional redundancy and flexibility in codon recognition. Similar patterns have been reported in other Rhodymeniales species, where anticodon diversity may improve translational efficiency and adaptability [9]. Variation in tRNA gene number and anticodon composition likely reflects lineage-specific fine-tuning of mitochondrial translation.

Phylogenetic analysis further clarifies the evolutionary placement of *R. intricata*. The topology recovered here is consistent with previous mitochondrial-based reconstructions [12]. Rhodymeniales formed a strongly supported monophyletic group, resolving into three main families: Rhodymeniaceae, Lomentariaceae, and Champiaceae. *R. intricata* clustered with its congeneric species *R. pseudopalmata* with high bootstrap support, confirming its taxonomic placement within Rhodymeniaceae. Lomentariaceae appeared as the earliest diverging lineage, followed by Champiaceae, indicating an early split among these families. This pattern agrees with earlier mitochondrial and multigene phylogenies [11,31].

Overall, the mitogenome of *R. intricata* combines conserved features typical of Rhodymeniales with lineage-specific gene content variation. The presence of additional genes and ORFs, together with consistent phylogenetic placement, highlights the dynamic yet structured evolution of mitogenomes in this order.

## 5. Limitations and Future Perspectives

This study provides key insights into the mitogenome of *R. intricata*, but several limitations remain. The mitogenome was assembled from a single specimen, so features such as the absence of *trnI*, TTG start codons, presence of *rns5*, and *orf148* may not capture intraspecific variation. Environmental influences and functional validation were not assessed, and the findings rely primarily on in silico analyses. Future work is needed to include transcriptomic/proteomic validation, nuclear and chloroplast genome sequencing, and population-level sampling across diverse regions to clarify organellar variation, ecological adaptation, and evolutionary patterns within Rhodymeniales.

## 6. Conclusions

In this study, the complete mitogenome of *R. intricata* was successfully sequenced, assembled, and analyzed, providing new genomic information for the order Rhodymeniales. The mitogenome is a circular DNA molecule of 26,213 bp and contains 49 genes, including 25 PCGs, 21 tRNAs, and 3 rRNAs. The genome shows a strong A + T bias, positive AT and GC skews, and a compact organization, which are characteristic features of red algal mitogenomes. Comparative analysis with other Rhodymeniales species revealed that the overall gene content and genome organization of *R. intricata* are largely conserved. However, several lineage-specific features were identified, including the presence of the *rpl20* gene, an additional ORF (*orf148*), and three rRNA genes (*rnl*, *rns*, and *rns5*). The absence of the *trnI* gene and the presence of multiple anticodon variants in some tRNAs further highlight the dynamic nature of mitogenome evolution within this group. Codon usage analysis showed a clear preference for certain amino acids, particularly leucine and isoleucine, as well as dominant start and stop codons (ATG and TAA), reflecting translational selection in the mitogenome. Phylogenetic analysis based on concatenated mitochondrial PCGs provides strong support for the monophyly of Rhodymeniales and clearly resolved relationships within its major families. *R. intricata* clustered closely with its congeneric species *R. pseudopalmata*, confirming its taxonomic position within the family Rhodymeniaceae. The topology obtained in this study is consistent with previous mitochondrial and multigene phylogenetic studies.

Overall, the mitogenome of *R. intricata* expands the currently available mitogenomic resources for Rhodymeniales and provides valuable insights into genome organization, gene evolution, and phylogenetic relationships within this group of red algae. These findings contribute to a better understanding of mitogenome evolution in Rhodophyta and provide a useful reference for future comparative and evolutionary studies of red algal mitogenomes.

## Figures and Tables

**Table 1:** List of the red algae species mitogenome sequences used in this study.

Species	Size (bp)	Total Genes	PCG	tRNA	rRNA	ORF *	A + T (%)	G + C (%)	Refs.
*Rhodymenia intricata* (PX733923)	26,213	49	24	21	3	1	70.9	29.1	In this study
*Rhodymenia pseudopalmata* (KC875852)	26,166	48	24	21	2	1	70.5	29.5	[30]
*Fushitsunagia catenata* (OR045827)	25,889	50	24	23	2	1	70.4	29.6	[12]
*Coeloseira compressa* (KU053956) *	25,391	45	24	19	2	-	74.3	25.7	[31]
*Acrosorium flabellatum* (PX111276)	31,818	61	24	33	2	2	78.8	21.2	-

Note: * indicates partial sequence of mitogenome.

**Table 2:** Features of the mitogenome of *Rhodymenia intricata* (GenBank accession number PX733923).

Gene Name	Start	End	Length (bp)	Amino Acids	Start Codon	Stop Codon	Anti-Codon	Strand	Gap/Overlap
*rnl*	1	2654	2654	-	-	-	-	H	0
*rps3*	2667	3353	687	228	ATG	TAA	-	H	12
*rpl16*	3353	3757	405	134	ATG	TAA	-	H	−1
*trnD*	3769	3840	72	-	-	-	GTC	H	11
*cox1*	3889	5481	1593	530	ATG	TAA	-	H	48
*cox2*	5530	6294	765	254	TTG	TAG	-	H	48
*cox3*	6437	7255	819	272	ATG	TAA	-	H	142
*atp4*	7257	7799	543	180	ATG	TAA	-	H	1
*trnG*	7800	7873	74	-	-	-	TCC	H	0
*trnQ*	7882	7954	73	-	-	-	TTG	H	8
*trnL*	7981	8065	85	-	-	-	TAA	H	26
*cob*	8108	9250	1143	380	ATG	TAA	-	H	42
*trnL*	9537	9621	85	-	-	-	TAG	L	286
*nad6*	9623	10,231	609	202	ATG	TAA	-	L	1
*trnG*	10,257	10,328	72	-	-	-	GCC	L	25
*trnH*	10,341	10,415	75	-	-	-	GTG	L	12
*sdh2*	10,417	11,166	750	249	ATG	TAG	-	L	1
*sdh3*	11,179	11,559	381	126	ATG	TAA	-	L	12
*trnF*	11,587	11,660	74	-	-	-	GAA	L	27
*trnS*	11,670	11,753	84	-	-	-	TGA	L	9
*trnP*	11,764	11,836	73	-	-	-	TGG	L	10
*atp9*	11,874	12,104	231	76	ATG	TAA	-	L	37
*trnC*	12,146	12,218	73	-	-	-	GCA	L	41
*trnM*	12,226	12,299	74	-	-	-	CAT	L	7
*rps11*	12,309	12,665	357	118	TTG	TAA	-	L	9
*rns5*	12,686	12,792	107	-	-	-	-	L	20
*nad3*	12,807	13,172	366	121	ATG	TAA	-	L	14
*nad1*	13,178	14,158	981	326	ATG	TAA	-	L	5
*nad2*	14,170	15,657	1488	495	ATG	TAA	-	L	11
*sdh4*	15,680	15,919	240	79	ATG	TAA	-	L	22
*nad4*	15,924	17,399	1476	491	ATG	TAG	-	L	4
*nad5*	17,997	19,982	1986	661	ATG	TAG	-	L	597
*atp8*	19,997	20,401	405	134	ATG	TAA	-	L	14
*atp6*	20,423	21,181	759	252	ATG	TAA	-	L	21
*trnW*	21,210	21,281	72	-	-	-	TCA	L	28
*orf148*	21,316	21,762	447	148	ATG	TAA	-	L	34
*trnA*	21,789	21,862	74	-	-	-	TGC	L	26
*trnS*	21,909	22,002	94	-	-	-	GCT	L	46
*trnN*	22,532	22,607	76	-	-	-	GTT	H	529
*trnV*	22,623	22,695	73	-	-	-	TAC	H	15
*trnR*	22,704	22,779	76	-	-	-	ACG	H	8
*trnK*	22,795	22,868	74	-	-	-	TTT	H	15
*tatC*	22,891	23,625	735	244	TTG	TAA	-	H	22
*rps12*	23,627	23,995	369	122	ATG	TAA	-	H	1
*trnE*	24,005	24,077	73	-	-	-	TTC	H	9
*trnM*	24,079	24,152	74	-	-	-	CAT	H	1
*rpl20*	24,177	24,404	228	75	ATG	TAA	-	H	24
*rns*	24,460	25,847	1388	-	-	-	-	H	55
*nad4 L*	25,908	26,213	306	101	ATG	TAA	-	H	60

**Table 3:** Nucleotide composition of mitogenomes in four Rhodymeniales species.

Name	Length (bp)	Nucleotide Composition (%)						AT- Skew	GC-Skew
		A	T	G	C	A + T	G + C		
**Whole genome**									
*Rhodymenia intricata*	26,213	37.4	33.6	14.7	14.4	70.9	29.1	0.054	0.009
*Rhodymenia pseudopalmata*	26,166	36.9	33.6	15.0	14.5	70.5	29.5	0.046	0.016
*Fushitsunagia catenata*	25,889	37.1	33.3	15.1	14.5	70.4	29.6	0.055	0.020
*Coeloseira compressa*	25,291	39.7	34.6	12.9	12.8	74.3	25.7	0.069	0.002
**PCGs**									
*Rhodymenia intricata*	18,069	29.3	42.0	14.9	13.8	71.3	28.7	−0.179	0.036
*Rhodymenia pseudopalmata*	17,820	29.3	41.3	15.2	14.3	70.5	29.5	−0.170	0.033
*Fushitsunagia catenata*	17,955	29.5	41.1	15.3	14.2	70.6	29.4	−0.164	0.037
*Coeloseira compressa*	17,508	29.8	45.3	13.4	11.6	75.0	25.0	−0.207	0.071

**Table 4:** Nucleotide composition and skews of protein-coding genes in the mitogenome of *Rhodymenia intricata*.

PCG	Length (bp)	Nucleotide Composition (%)						AT-Skew	GC-Skew
		A	T	G	C	A + T	G + C		
*rps3*	687	36.2	38.4	11.2	14.1	74.7	25.3	−0.029	−0.115
*rpl16*	405	42.5	31.9	12.6	13.1	74.3	25.7	0.143	−0.019
*cox1*	1593	26.0	40.9	18.0	15.1	66.9	33.1	−0.223	0.087
*cox2*	765	28.2	38.4	17.4	15.9	66.7	33.3	−0.153	0.043
*cox3*	819	26.1	41.8	17.1	15.0	67.9	32.1	−0.230	0.065
*atp4*	543	37.6	39.8	9.8	12.9	77.3	22.7	−0.029	−0.138
*cob*	1143	28.3	41.6	15.9	14.2	69.9	30.1	−0.191	0.058
*nad6*	609	27.1	45.3	15.6	12.0	72.4	27.6	−0.252	0.131
*sdh2*	750	34.0	35.9	14.4	15.7	69.9	30.1	−0.027	−0.044
*sdh3*	381	28.6	46.7	10.8	13.9	75.3	24.7	−0.240	−0.128
*atp9*	231	26.4	38.1	20.8	14.7	64.5	35.5	−0.181	0.171
*rps11*	357	42.6	33.9	9.8	13.7	76.5	23.5	0.114	−0.167
*nad3*	366	26.0	49.2	15.0	9.8	75.1	24.9	−0.309	0.209
*nad1*	981	25.5	43.4	17.9	13.1	68.9	31.1	−0.260	0.154
*nad2*	1488	27.0	45.8	14.2	13.0	72.8	27.2	−0.258	0.045
*sdh4*	240	28.3	45.0	13.8	12.9	73.3	26.7	−0.227	0.031
*nad4*	1476	25.1	47.0	15.4	12.6	72.0	28.0	−0.304	0.099
*nad5*	1986	26.6	44.3	15.8	13.3	70.9	29.1	−0.249	0.087
*atp8*	405	35.8	39.0	11.6	13.6	74.8	25.2	−0.043	−0.078
*atp6*	759	28.6	42.2	13.6	15.7	70.8	29.2	−0.192	−0.072
*orf148*	447	32.7	38.7	13.6	15.0	71.4	28.6	−0.085	−0.047
*tatC*	735	28.3	47.3	10.9	13.5	75.6	24.4	−0.252	−0.106
*rps12*	369	38.5	28.2	15.4	17.9	66.7	33.3	0.154	−0.073
*rpl20*	228	38.6	38.2	11.8	11.4	76.8	23.2	0.006	0.019
*nad4 L*	306	32.4	44.1	14.1	9.5	76.5	23.5	−0.154	0.194

**Table 5:** Features of mitochondrial protein-coding genes in four Rhodymeniales species.

Gene	Length (bp)	Amino Acids	Start Codon	Stop Codon	Strand
*rps3*	687 ^a,b^/681 ^c^/675 ^d^	228 ^a,b^/226 ^c^/224 ^d^	ATG ^a,b,c,d^	TAA ^a,b^/TAG ^c,d^	H ^a,b,c,d^
*rpl16*	405 ^a,c,d^/411 ^b^	134 ^a,c,d^/136 ^b^	ATG ^a,b,c,d^	TAA ^a,b,c^/TAG ^d^	H ^a,b,c,d^
*cox1*	1593 ^a^/1596 ^b,c,d^	530 ^a^/531 ^b,c,d^	ATG ^a,b,c,d^	TAA ^a,b,c,d^	H ^a,b,c,d^
*cox2*	765 ^a^/762 ^b^/798 ^c^/783 ^d^	254 ^a^/253 ^b^/265 ^c^/260 ^d^	ATG ^b,c,d^/TTG ^a^	TAA ^b,c,d^/TAG ^a^	H ^a,b,c,d^
*cox3*	819 ^a,b,c,d^	272 ^a,b,c,d^	ATG ^a,b,c,d^	TAA ^a,b,c,d^	H ^a,b,c,d^
*atp4*	543 ^a,b,c^/549 ^d^	180 ^a,b,c^/182 ^d^	ATG ^a,b,c,d^	TAA ^a,c^/TAG ^b,d^	H ^a,b,c,d^
*cob*	1143 ^a,b,d^/1161 ^c^	380 ^a,b,d^/386 ^c^	ATG ^a,b,c,d^	TAA ^a,b,c^/TAG ^d^	H ^a,b,c,d^
*nad6*	609 ^a,b,c,d^	202 ^a,b,c,d^	ATG ^a,b,c,d^	TAA ^a,b,c,d^	L ^a,b,c,d^
*sdh2*	750 ^a,b,c^/759 ^d^	249 ^a,b,c^/252 ^d^	ATG ^a,b,c,d^	TAA ^b,c,d^/TAG ^a^	L ^a,b,c,d^
*sdh3*	381 ^a,b,c^/300 ^d^	126 ^a,b,c^/99 ^d^	ATG ^a,b,c,d^	TAA ^a,b,d^/TAG ^c^	L ^a,b,c,d^
*atp9*	231 ^a,b,c,d^	76 ^a,b,c,d^	ATG ^a,b,c,d^	TAA ^a,c,d^/TAG ^b^	L ^a,b,c,d^
*rps11*	357 ^a^/354 ^b,c,d^	118 ^a^/117 ^b,c,d^	ATG ^b,c,d^/TTG ^a^	TAA ^a,b,d^/TAG ^c^	L ^a,b,c,d^
*nad3*	366 ^a,b,c,d^	121 ^a,b,c,d^	ATG ^a,b,c,d^	TAA ^a,b,c,d^	L ^a,b,c,d^
*nad1*	981 ^a,b,c,d^	326 ^a,b,c,d^	ATG ^a,b,c,d^	TAA ^a,b,c,d^	L ^a,b,c,d^
*nad2*	1488 ^a,b^/1485 ^c^/1491 ^d^	495 ^a,b^/494 ^c^/496 ^d^	ATG ^a,b,c,d^	TAA ^a,b,c,d^	L ^a,b,c,d^
*sdh4*	240 ^a,c,d^/225 ^b^	79 ^a,c,d^/74 ^b^	ATG ^a,b,c,d^	TAA ^a,c,d^/TAG ^b^	L ^a,b,c,d^
*nad4*	1476 ^a,b^/1482 ^c^/1467 ^d^	491 ^a,b^/493 ^c^/488 ^d^	ATG ^a,b,c,d^	TAA ^b,c,d^/TAG ^a^	L ^a,b,c,d^
*nad5*	1986 ^a,c,d^/1941 ^b^	661 ^a,c,d^/641 ^b^	ATG ^a,b,c,d^	TAA ^b,c,d^/TAG ^a^	L ^a,b,c,d^
*atp8*	405 ^a,b^/411 ^c^/414 ^d^	134 ^a,b^/136 ^c^/137 ^d^	ATG ^a,b,c,d^	TAA ^a,b,c,d^	L ^a,b,c,d^
*atp6*	759 ^a,b,c^/762 ^d^	252 ^a,b,c^/253 ^d^	ATG ^a,b,c,d^	TAA ^a,b,c,d^	L ^a,b,c,d^
ORF	447 ^a,b^/480 ^c^	148 ^a,b^/159 ^c^	ATG ^a,b,c^	TAA ^a,b,c^	L ^a,b,c^
*tatC*	735 ^a^/771 ^b^/756 ^c^/729 ^d^	244 ^a^/256 ^b^/251 ^c^/242 ^d^	ATG ^c,d^/TTA ^b^/TTG ^a^	TAA ^a,c^/TAG ^b,d^	H ^a,b,c,d^
*rps12*	369 ^a,b^/375 ^c^/348 ^d^	122 ^a,b^/124 ^c^/115 ^d^	ATG ^a,b,c,d^	TAA ^a,b,c,d^	H ^a,b,c,d^
*rpl20*	228 ^a^/195 ^d^	75 ^a^/64 ^d^	ATG ^a,d^	TAA ^a,d^	H ^a,d^
*nad4 L*	306 ^a,b,c,d^	101 ^a,b,c,d^	ATG ^a,b,c,d^	TAA ^a,b,c,d^	H ^a,b,c,d^

Note: ORF-open reading frame; ^a^ *Rhodymenia intricata* (PX733923); ^b^ *Rhodymenia pseudopalmata* (KC875852); ^c^ *Fushitsunagia catenata* (OR045827); ^d^ *Coeloseira compressa* (KU053956).

## Data Availability

The genome sequence data that support the findings of this study are openly available in GenBank of NCBI at (https://www.ncbi.nlm.nih.gov/, accessed on 20 August 2026) under accession number PX733923. The associated BioProject, Bio-Sample and SRA numbers were PRJNA1390879, SAMN54208080, and SRR36514420, respectively.
